# Needle in the Larynx

**Published:** 1828-12

**Authors:** 


### NEEDLE IN THE LARYNX.

### *Case in which a Needle was introduced into the Larynx.*

A MAN had been using a needle for the purpose of scratching his nostril: having let it go, it passed backwards into the fauces, and fell into the windpipe. The needle had a thread attached to it, which was entirely drawn in, and disappeared. Violent fits of coughing and attempts at expectoration immediately came on: by these the end of the thread was ejected, and the patient laid hold of this and pulled it. These attempts gave him great pain, but were unavailing. He continued for three days in a state of great anxiety and suffering, during which he made numerous ineffectual attempts to pull out the needle. At length he came to the BEAUJON, at Paris.

The thread was still hanging out of the mouth, and some efforts were again made by the house surgeon to extract the needle by pulling it gently, but in vain. M. BLANDIN, when he arrived, found that the thread had disappeared during the act of deglutition, nor could he recover it by introducing the fingers into the pharynx, nor by any other means. Uncertain whether the needle had really got into the larynx or the gullet, he contented himself with applying thirty leeches to the throat, followed by a poultice, &c. Next day the patient was much in the same state, and was bled to sixteen ounces, and had twenty leeches to the neck, &c.

For two days more there was little to remark; when, during the visit, the patient expelled the end of the thread in a fit of coughing. M. Blandin, having satisfied himself that the needle could not be pulled out, fixed the thread upon the cheek with a little adhesive strap, and resolved to operate next day.

On the following morning the respiration was more difficult, and the voice more hoarse. M. Blandin, having again tried various means of extracting the needle, proceeded to operate. The patient was placed horizontally on a bed facing the light, and M. Blandin, standing on the right side of the patient, fixed the larynx with the left hand, and then endeavoured to find the crico-thyroidean space, but the swelling rendered this impossible; he therefore made an incision through the skin on the median line, about a third of the length of the throat, and afterwards divided the subjacent parts very cautiously: it was not till he had penetrated to the depth of an inch that he laid bare the crico-thyroid membrane. Some bleeding took place, but the hemorrhage soon ceased. The nail of the forefinger of the left hand was placed transversely on the membrane, which was then punctured, and cut in the same direction. A grooved and curved director was introduced by the wound, and carried upwards, and the thyroid cartilage divided upon it throughout its whole length. Respiration was now freely performed through this large opening, but the voice was lost. A polypus forceps was introduced at two different times, and speedily withdrawn, on account of the irritation it excited, but without the needle. Considering it possible that the needle might be expelled in a fit of coughing, the patient was put to bed, the wound being lightly covered with a piece of linen pierced with holes, and spread with cerate.

On the following day a needle, nineteen lines in length, blackened, and as it were bronzed, was found attached to the compress laid over the wound.

The wound healed very slowly. The operation was performed on the 22d of June, and a fistulous opening, with great weakness and hoarseness of voice, remained in September. On the 30th of that month, it is stated that, by means of caustic applied to the edges of the aperture, it had at length closed, and the voice regained some of its former strength.*

